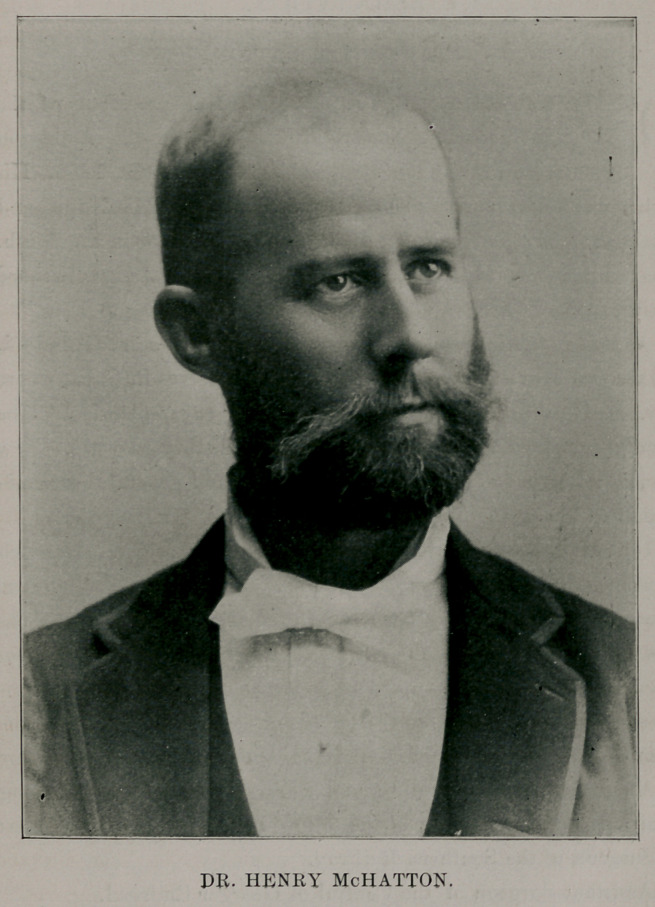# Dr. Henry McHatton

**Published:** 1903-06

**Authors:** 


DR. HENRY McHATTON.

### THE NEW OFFICERS OF THE GEORGIA MEDICAL ASSOCIATION.

### DR. HENRY McHATTON.

DR. HENRY McHATTON, the newly elected president of the Georgia Medical Association, was born on the Arlington plantation near Baton Rouge, La., February 29, 1856. His father was an extensive planter in Louisiana and Mississippi, and established the first cotton mill in Louisiana, if not in the South. His mother was Miss Eliza Chinn, daughter of Judge Richard Chinn of New Orleans, and authoress of "From Flag to Flag."

As the war progressed the family refugeed through Mississippi, Texas and Mexico, finally establishing themselves in Cuba, where Mr. McHatton resumed his occupation of sugar-planting. The family remained in Cuba until 1877, when they moved to New York on account of the long protracted war and general disorganization of affairs in Cuba.

Dr. McHatton secured his classical education in Norwich Academy, Norwich, Conn., and his medical education in Bellevue Hospital Medical College, where he graduated in 1881, one of the few members of his class that took the three years' course by election. He spent nearly two years in special medical studies in New York, and began the practice of his profession in Macon in November, 1882; since which time he has been a constant contributor to current medical literature, and at present holds the following positions, and is a member of the following scientific organizations:

Surgeon of the Southern Railway.

Assistant Surgeon of the Central of Georgia Railroad.

Chairman of Medical Board and Visiting Surgeon to Macon Hospital.

Member of the American Medical Association.

Member of National Association of Railroad Surgeons.

Member of Southern Association of Railroad Surgeons.

Member of National Academy of Railroad Surgeons.

Member of Pan-American Medical Association.

Member of National Congress of Tuberculosis.

Member of Medico-Legal Society of New York.

Member of American Association for the Advancement of Science.

Member of American Ornithological Union.

Honorary member of the King's County Medical Association of New York.

Ex-president Macon Medical Society.

Dr. McHatton married Miss Eliza Hubbard, of Norwich, Conn. They have one son, T. H. McHatton.

## Figures and Tables

**Figure f1:**